# Experiences of receiving an mHealth application with proactive nursing support among community-dwelling older adults: a mixed-methods study

**DOI:** 10.1186/s12912-024-01909-w

**Published:** 2024-04-07

**Authors:** Arkers Kwan Ching Wong, Jonathan Bayuo, Frances Kam Yuet Wong, Karen Kit Sum Chow, Siu Man Wong, Bonnie Bo Wong, Khloe Hau Yi Law

**Affiliations:** 1 School of Nursing, The Hong Kong Polytechnic University, 1 Cheong Wan Road, Hung Hom, Kowloon, Hong Kong China; 2 Hong Kong Lutheran Social Services, Homantin, Kowloon, Hong Kong China

**Keywords:** Community-dwelling older adults, mHealth, Mobile applications

## Abstract

**Background:**

As the population ages, a plethora of digital and mobile health applications for assistance with independent living have emerged. Still unknown, however, is how older adults sustain the use of these applications.

**Aim:**

This study sought to explore the experiences of older adults following their participation in a programme that combined the use of an mHealth application with proactive telecare nursing support.

**Methods:**

We employed a concurrent mixed-methods design for this study. The quantitative strand included a survey, whereas the qualitative strand included open-ended questions as part of the survey to understand the participants’ experiences. Participants for this study were community-dwelling older adults who had taken part in an interventional study that sought to examine the effects of mHealth and nurse support. A convenience sampling approach was employed to recruit potential participants for this study.

**Findings:**

: Fifty-five older adults participated. The majority expressed positive attitudes and satisfaction with the app and the nurses’ support. The app and nurses’ support helped participants to understand their health status and obtain health information. Reasons to halt app usage included technical issues and limited social support.

**Conclusion:**

Mobile apps with professional follow-up support could potentially support older adults in the community, although emerging concerns need to be addressed to sustain long-term usage of these apps.

**Supplementary Information:**

The online version contains supplementary material available at 10.1186/s12912-024-01909-w.

## Background

Technological advancements and a high smartphone penetration rate have enabled mobile health applications (mHealth apps) to be introduced to promote health awareness and self-management among community-dwelling older adults. mHealth apps commonly include features such as ongoing monitoring of vital signs and symptoms, the delivery of multimedia health information, medication alarm functions, and follow-up appointment notifications [1], which have been proven to be effective at enhancing the self-care self-efficacy [2–3] and quality of life [4] of older adults, and at reducing avoidable health service utilizations [5]. However, despite the many benefits attributed to mHealth apps, studies have not demonstrated enhanced and sustained usage among older adults. Among the different age groups, older adults remain the least likely to use mHealth apps [6]. Data from the United States suggests that only 38% of people over the age of 65 have used mHealth apps to monitor their health [7]. Among those who installed mHealth apps, less than 30% demonstrated actual use [7]. Also, 51% of older adults abandoned the use of mHealth apps within one year, as their interest or motivation to use them declined [8–9].

Some studies have suggested that the perceived difficulty of usage and perceived lack of usefulness as the two chief barriers that may hinder the engagement and motivation of older adults to adopt mHealth apps in the long term [10, 11]. Some older adults may find mHealth apps difficult to use due to a decline in their cognitive abilities [11]. With limited short-term memory and a slower processing speed, the increased likelihood that they will experience memory overload could slow down their initial ability to utilize the app, such as for filling in information [11]. With visual and cognitive degeneration, it is likely that older adults would feel overwhelmed when presented with new and lengthy health information. They may also find mHealth apps incompatible with their lifestyle and unreliable to use [10]. Additional barriers identified in existing literature include lack of knowledge regarding how to use mHealth, decreased sensory perception, lack of need for technology, poorly designed interface, cost of technology, and limited/fixed income [11].

Some older adults consider themselves competent and independent enough to develop strategies for self-care and are therefore unable to see the advantages of or the need to use mHealth for assistance [12]. They have also suggested that the data measurements in mHealth apps are often inaccurate or inconsistent, and have expressed doubt about relying on the health information in the apps, with unspecified sources and medical abbreviations, without the support of professionals [13–14].

Older adults may also have negative attitudes toward the use of mHealth apps in self-management due to technological anxiety. Technological anxiety is a feeling of uneasiness and fear about using technology, and this doubt about their ability to operate technology properly may lower the self-efficacy of older adults [15]. Older adults with poor technological knowledge would perceive mHealth apps as being not very effective, and would be especially doubtful about the ability of such apps to generate accurate measurements [16]. They might transfer their mistrust towards technology to mHealth apps, regarding such apps as being at risk of faulty technology and frequent updates [17].

In order to help older adults tackle the barriers to using mHealth apps and to facilitate their sustained usage of such apps so as to enhance their health and well-being, it is crucial to have a healthcare professional who can provide guidance and individualized consultations on the use of such apps when necessary. A nurse case manager may have the ability to motivate and help older adults to enhance and sustain their usage of these apps, and could provide professional medical knowledge by delivering timely feedback regarding the inputted vital signs in the apps, which older adults have found useful and engaging [18–19]. Cajita and her colleagues [20] have suggested that older adults feel secure when nurses monitor their health via the apps and provide individual suggestions if abnormal readings are discovered. The involvement of the same nurse could further allow consistent treatment planning and the building of a long-term therapeutic relationship with older adults. In addition, the nurse case manager can rely on some features of the mHealth app, such as the health education corner, online communication platform, and symptom checker, to empower older adults to engage in shared decision-making, goal setting, formulating an individualized treatment plan, and evaluating their progress from time to time which, taken together, would promote the use of mHealth apps by older adults over the long term [21].

With the aim of facilitating the long-term use of mHealth apps among community-dwelling older adults, our research team developed a programme that incorporates the services of a nurse case manager in the design and operation of the app [22–25]. The study divided the older adults into three groups: the mHealth group, whose participants were allowed to use an mHealth app independently; the mHealth + I group, whose participants received eight proactive calls in three months from a nurse case manager to encourage use of the app; and the control group, whose participants received neither an mHealth app nor support from the nurse case manager. The application offers various functionalities, which include monitoring vital signs, scheduling appointments, notifying about medications, and providing up-to-date health education. In the mHealth + I group, a nurse regularly monitored participants’ vital signs in the app’s database. Whenever any abnormalities were detected, the nurse would contact the participants within 24 h via smartphone. Following the established protocol, she would assess the participants and provide education on self-care techniques and knowledge, or refer them to a hospital if necessary. The app was used on daily basis throughout the 12-week period. Following this, another 12-week period was set to ascertain the sustained effects of the combined intervention. The study observed that participants in the mHealth group demonstrated improvement in the Physical Component Summary (PCS) of the Quality-of-Life scale over the 12-week period and sustained to the 12-week follow-up period whereas the mHealth + I group demonstrated improved self-efficacy over the intervention period with a decrease at follow-up [23]. These beneficial effects notwithstanding, it is interesting to note that the mHealth + I group that received more support experienced minimal improvement which warrants a further study to ascertain how they experienced the use of the mHealth app and the proactive professional support. The aim of this paper is to explore the experience of the mHealth + I group of participants in using the mHealth app with the proactive support of nurse case manager. If the programme receives positive feedback, it could act as a blueprint for a future study on motivating older adults to continue to use mHealth apps and to maximize the effect of the apps on health promotion and self-management.

## Materials and methods

### Study design

A convergent mixed-methods approach was used in this study [26]. This approach involved the simultaneous collection of quantitative and qualitative data in a single study. The reason for adopting this approach was to obtain various kinds of data to understand how participants experienced the mHealth app and nurse follow-up service. The quantitative component included a cross-sectional survey, whereas the qualitative component employed a qualitative descriptive approach with open-ended questions to understand the participants’ experiences and help explain the data obtained from the quantitative strand.

### Participants and recruitment

Participants for this study were community-dwelling older adults who had taken part in an interventional study that sought to examine the effects of mHealth and nurse support [23]. The participants were recruited from five community and elderly centers that serve more than 50,000 older adults per year. Older adults with at least one of the following problems– hypertension, diabetes, or chronic pain– were recruited to the programme if they (1) were aged 60 or above and (2) had a smartphone. Excluded were those who (1) had already been involved in other mHealth programmes, (2) had been hospitalized with a known psychiatric problem within the last six months, (3) were bedbound, or (4) did not have internet coverage at home. Following the completion of the intervention, participants in the mHealth + I group were invited to participate after a one-week period to minimise recall bias. Those who expressed interest were assigned to a research associate to complete the processes of recruitment and data collection.

### Sampling and sample size

A convenience sampling approach was employed to recruit participants for this phase of the study. Staff in the community and elderly centers called potential eligible participants from their membership list, confirmed their eligibility, explained the program to them, and sought their consent to participate. Our goal was to recruit all older persons who had received the mHealth and nurse support intervention (*n* = 74), although some participants declined to participate.

### Instrumentation and data collection

A pre-designed questionnaire in Cantonese was used to obtain data from the participants (See supplementary file). The questionnaire was designed solely for this study and was comprised of three questions focusing on attitudes to mHealth; five questions focusing on satisfaction with mHealth, and four open-ended questions to capture the participants’ experiences. The instrument was piloted among a group of older adults prior to the collecting of data. Minor changes were made to the text to improve understanding after the pilot. The first two components were in the form of a Likert scale with responses ranging from 1 (strongly disagree) to 5 (strongly agree). The final version was uploaded to Qualtrics, an online survey tool utilized for collecting feedback from the participants, and subsequently distributed to them. Older adults who agreed to participate were sent a secure link to allow them to access and complete the instrument. A research associate was assigned to assist participants who had difficulties in accessing the instrument. All responses were securely maintained in Cloud storage and were accessible only to the members of the research team.

### Ethical considerations

Ethical approval for the study was obtained from the Ethics Sub-committee of the Hong Kong Polytechnic University (HSEARS20190312002). All methods regarding the study were performed in accordance with the ethical guidelines regarding human research of the institution. All of the participants gave their written consent to participate in the study. Informed consent was obtained from all participants prior to their inclusion in the study. They were assured that they could withdraw from the study at any time without any adverse consequences. The collected data were pseudonymized, encrypted and stored in the Qualtrics.

### Data analysis

Following data collection, the populated file was downloaded in the form of an Excel file. The file was reviewed for completeness, following which two team members fluent in both Cantonese and English translated the responses into English. The final version was reviewed and approved by the lead author. The quantitative dataset was exported to SPSS version 29 and analysed using descriptive statistics in the form of tables. Conventional content analysis was employed for the open-ended questions or qualitative strand [27]. The analysis was undertaken in both Cantonese and English, and the authors compared the two versions to ensure that the original meanings were retained. This was done by formulating codes across all the response obtained from the open-ended questions. To do this, two authors reviewed all the statements independently in Microsoft Excel to formulate initial codes. These codes were discussed with the research team to formulate an initial coding frame. The coding frame was applied to the responses while refining the frame further. This continued until all the responses were exhausted. Following these, the codes were aggregated and reviewed by the team. Similar codes were then aggregated to formulate sub-categories. These were reviewed alongside the survey findings which helped formulate categories. Following a separate analysis of the two datasets, the findings were integrated by examining how both datasets were related, and in response to the overall study aim (see Tables 1 and 2).

## Results

### Socio-demographic characteristics

Fifty-five out of 74 participants agreed to join the programme and completed the questionnaire. Reasons for declining participation in the program included busy (*n* = 18) and admitted to hospital (*n* = 1). The majority of them was female (85%). The mean age was 66 with standard deviation of 7.23. The majority of them lived in a flat (90%) alone (33%), or with family members (55%) or a spouse only (12%). More than half of them have received primary or secondary educational level (91%), only few of them had no formal education before (9%). Participants were living with either chronic pain (20%), hypertension (40%), diabetes (10%), a combination of hypertension and diabetes (10%), and a combination of pain and hypertension (20%).

### Attitudes toward using the mHealth application

Regarding the attitudes of the participants towards the utilization of the mHealth apps (see Table 1), it emerged that up to 27% (*n* = 17) were positive about using the app, felt that the app could fit their needs, and thought that they would use the app in later years. Participants who disagreed or indicated that they were neutral noted that although the app was potentially helpful, either they did not know how to operate it (“The program design is good, but I don’t know how to use it myself, so it does not help”, P008) or felt that their condition did not warrant the use of the mHealth app: (“I usually have no special ailments, so I don’t need to use it”, P015), or felt they were already satisfied with how they were self-managing their condition without the use of the app (“Because I know how to manage, I don’t need help”, P022).

Those participants who agreed that they liked the app singled out the following special features as being particularly notable: (i) the general health information, which allowed them to learn on their own even in the absence of a healthcare professional (“You can find out information about a healthy diet and exercise from it, and then learn by yourself”, P002); (ii) the opportunity to interact with a nurse when required (“You can read health information and have telephone interviews with nurses to understand your health status”, P029); (iii) the ability to monitor their own health status regularly, thereby developing a healthy lifestyle (“Encourage yourself to measure your blood pressure and body temperature every day, and you can follow the health information to adjust your living habits”, P043); and (iv) the ability to do all of these things on their own and at their own convenience (“You can quickly know about changes in your health status. In addition, I think the most important thing is to be able to operate it by yourself, otherwise it is meaningless”, P033).

To these participants, the app could fit their needs by helping them to understand and monitor their health status as well as to take measures to live a healthier life with adequate follow-up by the nurses (“Learn more about health information and find ways to improve your health”, P006). Some participants agreed that they would use the app in later years, as the app had the capacity to store their health information, permitting retrieval at a later date (“It lets you keep your blood pressure status, which is convenient for you to provide when you see a doctor”, P001); however, they were also worried about potential memory loss, which could affect their use of the app (“…but you are worried that your memory will deteriorate in late life”, P019).

**Table 1:** Attitudes towards using the mHealth application

Variable		n	%	Qualitative categories
I like using this app	Strongly disagree	1	1.6	• Perceived limited usability and challenges with navigating through the app
	Disagree	11	17.5	
	Neutral	8	12.7	
	Agree	32	50.8	• Positive views regarding app design and usability
	Strongly agree	2	3.2	
Overall, I think this mobile app does just the right thing	Strongly disagree	0	0	• Perceived limited usability of the mHealth app
	Disagree	11	17.5	
	Neutral	14	22.2	
	Agree	30	47.6	• Health education and health promotion
				• Convenience and opportunities for self-management
				• Availability of professional support when required
Using this mobile app in my later years is ideal	Strongly disagree	0	0	• Limited app usability in later years
	Disagree	8	12.7	
	Neutral	30	47.6	
	Agree	16	25.4	• Opportunities for ongoing self-monitoring
	Strongly agree	1	1.6	
				• Potential long-term storage of vital statics/ health information

### Satisfaction with using the mHealth app and proactive nurse support

As shown in Table 2 below, the majority of participants were neutral regarding their satisfaction with using the app. Despite this, more participants agreed that they were satisfied with using the app compared to participants who disagreed. Participants agreed that their health had improved after using the app because the platform gave them an opportunity to monitor their health status, record vital parameters, and have them reviewed by a nurse who would take action if required (“When the nurse sees that the health indicators are not ideal, there will be a notification, so that you can pay more attention”, P007). This would allow them to self-manage their condition (“It is all right, when you encounter health problems, you can search on the programme to enhance your self-management ability”, P027). For participants who disagreed or were neutral, it was noted that their main concern was related to uncertainty about the ability of the mHealth app to support them. They were unsure about the functionality of the app and coupled with the fact that some of the participants had challenges with navigating through the app, they could not view the app as helping to improve their health (“I did not know how to use it myself, so it did not help me”, P043).

Regarding the time saved from having to search for health-related information, some participants felt that the app gave them the luxury to obtain information whenever they needed to (“There is a wealth of information, and you can find some health information to help you learn”, P048). Other participants who disagreed felt that the difficulty of navigating the app made it rather difficult for them to search for health-related information (“If you know how to use it, it can help you to find health information and then learn, but unfortunately I don’t know how to use it”, P017). In addition to the satisfaction expressed towards the app, some participants provided positive remarks regarding the support they received from the nurses. One participant mentioned the instructions provided by the nurses that helped to maintain healthy dietary habits: (“Yes, she [the nurse] reminded me to watch what I eat and avoid certain things”, P023). For some participants, the support provided by the nurses was central to maintaining healthy behaviours and was challenging disengaging with the nurses at the end of the project: (“At that time, I was a little disappointed when the nurse said she didn’t need to talk to me and that I didn’t have to find her. So, I said thank you and appreciated her calling me to talk. If there is such an opportunity in the future, I will participate, and I hope there will be more nurses to follow up. This way, I think it will be better for me personally”, P039). Regarding the alleviation of health-related concerns and improvement in self-care ability, some participants mentioned that having a nurse at the receiving end who can provide feedback via telephone calls on their parameters was helpful for monitoring and keeping track of their health status (“A telephone interview with the nurse helps one to understand one’s condition”, P020). For participants who disagreed or were neutral, the difficulty in navigating the app served as a major deterrent to avoid using the app as much as possible (“if you don’t know how to use it, it is meaningless”, P051).

Using open-ended questions, the participants were further asked about how they coped when they experienced discomfort while using the app. The responses included reaching out to the nurses for support (“Look up information and ask the nurses when there is a telephone interview”, P030); using over-the-counter medications (“There are few problems, and most can be solved by taking over-the-counter medicines”, P028); or going to the hospital for a doctor’s visit (“I ask the doctor directly during a visit”, P011). Regarding the participants’ plan to discontinue the app within six months and the reason for that, some participants mentioned that the busy nature of their work may force them to stop using the app (“Due to busy housework, I’m unable to take the time to use it”, P021). Others indicated that in the absence of their usual social support, they may find it difficult to use the app (“I don’t know how to use it. I have to rely on my daughter’s help, so it is useless if she is not around”, P036).

**Table 2:** Satisfaction with using the app

Variable		n	%	Qualitative categories
Health has improved after using the mobile application	Strongly disagree	1	1.6	• Uncertainty about app functionality
	Disagree	7	11.1	
	Neutral	26	41.3	
	Agree	20	31.7	• Opportunities for self-monitoring and self-management
	Strongly agree	1	1.6	
				• Follow-up professional support
Can save time spent searching for health-related information	Strongly disagree	0	0	• Perceived limited app functionality and challenges with navigating through
	Disagree	15	23.8	
	Neutral	26	41.3	
	Agree	14	22.2	• Convenience
				• Ease in obtaining health-related information
Can help to alleviate concerns related to health	Strongly disagree	1	1.6	• Perceived limited app functionality
	Disagree	15	23.8	
	Neutral	22	34.9	
	Agree	17	27	• Availability of professional follow-up support
	Strongly agree	0	0	
Can help to improve self-care ability	Strongly disagree	2	3.2	• Perceived limited app functionality
	Disagree	7	11.1	
	Neutral	30	47.6	
	Agree	16	25.4	• Opportunities for self-monitoring and self-management
				• Information seeking
				• Availability of professional follow-up support
Overall, how satisfied are you with using this mobile application?	Strongly disagree	1	1.6	• Dissatisfied due to perceived limited app functionality and challenges
	Disagree	11	17.5	
	Neutral	30	47.6	
	Agree	13	20.6	• Satisfied due to the app’s ability to support self-management, monitoring, and ongoing interaction with nurses

## Discussion

With an ongoing ageing population, older people now have a plethora of digital health tools and mobile health applications to assist them in living independently and self-managing their chronic ailments. The study sought to explore the experiences of older adults following their participation in a programme that provided proactive nursing support with the use of an mHealth application. Overall, the findings were mixed with regard to the participants’ attitudes, satisfaction, and experiences with the mHealth app and follow-up support delivered by the nurse case managers. Despite this, the findings shed light on what worked and provide a basis for improving the programme, such as by providing continuing education on how to use the app for those older adults who might need more time to adjust to it, and encouraging use of the app as way of promoting health for all older adults.

The mHealth app was generally noted to facilitate self-monitoring, the keeping of health records, access to health information, and follow-ups by a nurse case manager. This was a comprehensive way to help community-dwelling older adults navigate through their health needs while receiving professional support if required. The added component of nurse support was important, as it ensured that any deviations from their health parameters were detected and acted upon in a timely manner through follow-up interviews. The availability of follow-up support from the nurses was stressed by the participants, and some even mentioned that the nurses would be the first point of call if they experienced discomfort. Previous studies have reported that a major barrier to the utilization of mHealth apps was that healthcare professionals lacked the time to monitor and review the information obtained from the patients [28]. In the current study, this problem was overcome by ensuring that a nurse case manager was always available to monitor and provide feedback to the patient. In fact, although ageing is not a disease, it can lead to various health-related issues that warrant ongoing monitoring and professional input. The approach employed in this study mimics a process where an older adult and a nurse work collaboratively to improve health outcomes while encouraging independence and self-management as far as possible. However, future studies may be needed to explore the experiences of older adults who did not receive professional support, to ascertain whether their experiences might vary from those who did receive professional support.

Among the key issues worth highlighting here was the inability of participants to use or operate the app on their own and the notion of some that their condition did not require the use of the app, or that their condition was not severe enough to warrant the use of the app. Usability issues, including challenges with understanding the structure for navigating mHealth apps, such as text, buttons, and icon elements, have been reported in previous studies [29–30]. Thus, it has been recommended that these mHealth apps take into consideration the diminishing cognitive skills and physical abilities of older adults. In the current study, the community-dwelling older adults were trained in the use of the apps. However, some older adults may need more training or continual training to be able to use the app to boost their self-efficacy and self-confidence [31]. To improve utilization of mHealth apps, it may therefore be necessary to identify and support those older adults who may need more training. It may also be necessary to devise tailor-made continuing education programmes to address the unique needs of older adults.

The notion of disabling the app within six months is another concern worth mentioning. The older adults provided several reasons for wanting to disable the app, including technical issues with their phone/app, work schedule, challenges with operating the app, and the unavailability of social support. These findings suggest that sustained use of the app will be potentially problematic. In fact, studies have shown that user commitment to mHealth is generally low [32–33] with one study reporting that approximately 53% of such apps were uninstalled within 30 days of download, and that this may be related to factors such as a lack of interest [34]. One study highlighted the point that some older adults may feel that the app does not meet their expectations, which can lead to a failure to fulfil the requirements of using the app and, subsequently, to abandonment [35]. Perhaps, it would be beneficial to prioritize regular updates of the app, along with providing ongoing education for both middle-aged adults and older adults. This approach acknowledges that middle-aged adults who are already familiar with mobile apps will find it easier to transition into older adulthood, thereby fostering the acceptance and widespread use of mHealth apps.

### Study limitations

This study presented mixed findings regarding the participants’ experiences with the mHealth app and nurse follow-up support, although unique insights were provided to support further work. A notable limitation of this study is that the data were obtained following the completion of the intervention. Thus, there was a potential for recall bias among the older adult participants. Also, the use of open-ended questions instead of face-to-face interviews may have led to limited information regarding how the older adults experienced the mHealth app. Future studies may therefore require a more in-depth approach to data collection to attain a comprehensive understanding of the experiences of community-dwelling older adults. Another limitation worth considering is the fact that only older adults who had received mHealth and nurses’ support were considered eligible for this study which may limit the transferability of the study findings. For instance, older adults who received only the mHealth app may have experienced the phenomenon in a different manner and some findings from this study may not apply to them. The lack of a comparative group also remains another limitation of this study.

## Conclusion

Digital and mHealth applications have the potential to support continuity of care for community-dwelling older adults, although several factors need to be taken into consideration regarding their use over both the short term and long term. Ongoing education is needed to improve the self-efficacy and knowledge of older adults in navigating these apps, which may have the potential to lead to sustained use of these apps.

### Electronic supplementary material

Below is the link to the electronic supplementary material.

Supplementary Material 1

## Data Availability

All data generated or analysed during this study are included in this manuscript.
