# Supplementary material for: Experiences of receiving an mHealth application with proactive nursing support among community-dwelling older adults: a mixed-methods study
Source: BMC Nurs. 2024 Apr 7;23:232. doi: 10.1186/s12912-024-01909-w (PMC10999086; doi:10.1186/s12912-024-01909-w)
Supplement: Supplementary file 1 — Supplementary Material 1 [file 12912_2024_1909_MOESM1_ESM.pdf]

HONG KONG LUTHERAN SOCIAL SERVICE
LUTHERAN CHURCH - HONG KONG SYNOD

THE HONG KONG POLYTECHNIC UNIVERSITY

Questionnaire on "mHealth Application with proactive nursing support"

(A)	Attitudes towards using the mHealth application	Strongly agree	Agree	Neutral	Disagree	Strongly disagree
1.	I like using this app	5	4	3	2	1
2.	Overall, I think this mobile app does just the right thing	5	4	3	2	1
3.	Using this mobile app in my later years is ideal	5	4	3	2	1

(B)	Satisfaction with using the mHealth application	Strongly agree	Agree	Neutral	Disagree	Strongly disagree
1.	Health has improved after using the mobile application	5	4	3	2	1
2.	Can save time spent searching for health-related information	5	4	3	2	1
3.	Can help to alleviate concerns related to health	5	4	3	2	1
4.	Can help to improve self-care ability	5	4	3	2	1
5.	Overall, how satisfied are you with using this mobile application?	5	4	3	2	1
(C)	How does the app help you manage your health?					
(D)	How do you cope when you experienced discomfort while using the app?					
(E)	(If you disabled the app within 6 months, please answer the following questions)<br>Why do you stop using the app?					
(F)	Do you agree sharing your experiences on participating this program in a press? ☐ Agree ☐ Disagree<br>If you agree, please provide your contact information below:<br>Name： ____________ Tel： ____________ Center： ____________					
